# Optimizing Exogenous Surfactant as a Pulmonary Delivery Vehicle for Chicken Cathelicidin-2

**DOI:** 10.1038/s41598-020-66448-1

**Published:** 2020-06-10

**Authors:** Brandon Baer, Edwin J. A. Veldhuizen, Natalia Molchanova, Shehrazade Jekhmane, Markus Weingarth, Håvard Jenssen, Jennifer S. Lin, Annelise E. Barron, Cory Yamashita, Ruud Veldhuizen

**Affiliations:** 1 Department of Physiology and Pharmacology, Western University, London, Ontario Canada; 2 Department of Infectious Diseases and Immunology, Faculty of Veterinary Medicine, Utrecht University, Utrecht, The Netherlands; 3 Department of Science and Environment, Roskilde University, Roskilde, Denmark; 4 Division of Biological Nanostructures, The Molecular Foundry, Lawrence Berkeley National Laboratory, Berkeley, California USA; 5 Bijvoet Center for Biomolecular Research, Department of Chemistry, Utrecht University, Utrecht, The Netherlands; 6 Department of Bioengineering, School of Medicine & School of Engineering, Stanford University, Stanford, California USA; 7 Department of Medicine, Western University, London, Ontario Canada

**Keywords:** Peptides, Proteins, Lipids, Bacterial infection, Infectious diseases, Respiratory tract diseases, Drug delivery, Drug discovery and development, Pharmacology, Clinical pharmacology, Diseases, Solid-state NMR

## Abstract

The rising incidence of antibiotic-resistant lung infections has instigated a much-needed search for new therapeutic strategies. One proposed strategy is the use of exogenous surfactants to deliver antimicrobial peptides (AMPs), like CATH-2, to infected regions of the lung. CATH-2 can kill bacteria through a diverse range of antibacterial pathways and exogenous surfactant can improve pulmonary drug distribution. Unfortunately, mixing AMPs with commercially available exogenous surfactants has been shown to negatively impact their antimicrobial function. It was hypothesized that the phosphatidylglycerol component of surfactant was inhibiting AMP function and that an exogenous surfactant, with a reduced phosphatidylglycerol composition would increase peptide mediated killing at a distal site. To better understand how surfactant lipids interacted with CATH-2 and affected its function, isothermal titration calorimetry and solid-state nuclear magnetic resonance spectroscopy as well as bacterial killing curves against *Pseudomonas aeruginosa* were utilized. Additionally, the wet bridge transfer system was used to evaluate surfactant spreading and peptide transport. Phosphatidylglycerol was the only surfactant lipid to significantly inhibit CATH-2 function, showing a stronger electrostatic interaction with the peptide than other lipids. Although diluting the phosphatidylglycerol content in an existing surfactant, through the addition of other lipids, significantly improved peptide function and distal killing, it also reduced surfactant spreading. A synthetic phosphatidylglycerol-free surfactant however, was shown to further improve CATH-2 delivery and function at a remote site. Based on these *in vitro* experiments synthetic phosphatidylglycerol-free surfactants seem optimal for delivering AMPs to the lung.

## Introduction

The increasing incidence of antimicrobial resistance in bacterial pneumonia has instigated a much-needed search for new therapeutic approaches for these types of infections^1,2^. One approach, involving the utilization of exogenous surfactant for the delivery of antimicrobial peptides (AMPs) to the infected lungs is supported by a strong theoretical foundation^3,4^. The AMPs, such as the chicken cathelicidin, CATH-2, can target a wide spectrum of antibiotic-resistant bacteria, making them potential novel therapeutics for bacterial pneumonia, which can be delivered to the areas of infection by exogenous surfactant^3,5–7^. Unfortunately, simply mixing CATH-2 or other AMPs with a commercial exogenous surfactant has been shown to impact a drug’s therapeutic efficacy^5,8^. For example, we recently demonstrated that an exogenous surfactant with CATH-2, exhibited antimicrobial activity against antibiotic-resistant bacterial isolates from cystic fibrosis patients, albeit with less efficacy than CATH-2 by itself^5^. Therefore, in order to improve this promising approach, it is important to understand how surfactant interferes with cathelicidin function and investigate strategies to minimize this interaction, while maintaining the benefits of surfactant delivery.

The primary benefit of utilizing CATH-2, and other cathelicidins, for therapeutic purposes is their diverse range of pathways to kill bacteria since their positive charge allows them to interact directly with both the negatively charged lipids of the bacterial cell wall as well as intracellular targets such as DNA or RNA^9–12^. This multi-target approach to killing bacteria has been demonstrated to be effective against a wide spectrum of antibiotic-resistant organisms^5–7^. Unfortunately, the use of AMPs to treat bacterial lung infections has been largely unsuccessful, due to an inability to directly deliver these peptides to the peripheral sites of infection^13–15^. Therefore, improving the pulmonary delivery of these highly effective antimicrobial agents with an exogenous surfactant would have substantial therapeutic value.

The goal of using exogenous surfactant for drug delivery is to open up collapsed airways and areas with edema, in order for the therapeutic to reach the areas of the lung affected by infection^16,17^. Most commercially used exogenous surfactants are derived from animal lungs and are complex mixtures of phospholipids (85%), neutral lipids (5–8%) and specialized surfactant proteins, designated SP-B and SP-C (7–10%)^17–19^. Although variations exist among different products, the main lipid components of these exogenous surfactants are saturated (dipalmitoylphosphatidylcholine; DPPC, approx. 40%), unsaturated phosphatidylcholine (PC; approx. 35%), the negatively charged phosphatidylglycerol (PG; approx. 10%), and neutral lipids like cholesterol (5–8%)^3^. These lipids, together with SP-B and SP-C are ultimately responsible for the ability of exogenous surfactant to rapidly adsorb to the air-liquid interface and spread throughout the airways^18,20,21^.

Together, the above information on surfactant composition and cathelicidin properties indicates that CATH-2 may interact with the negatively charged phospholipids within surfactant. However, this has not been demonstrated directly, nor is there evidence that this interaction interferes with cathelicidin function or if this interaction is crucial for surfactant’s ability to act as a carrier for cathelicidins. It is hypothesized that the PG component of surfactant inhibits CATH-2 function and that an exogenous surfactant, with a reduced PG composition would increase peptide mediated killing at a distal site.

## Materials and Methods

### Preparations

The chicken cathelicidin, CATH-2 was synthesized and purified as described previously^22^. It is comprised of 26 amino acids - RFGRFLRKIRRFRPKVTITIQGSARF-NH2 and has a positive charge of +8^23^. The commercially available surfactant, bovine lipid extract surfactant (BLES) was generously provided by BLES Biochemicals (London, ON, Canada). BLES was stored in 100 mM sodium chloride and 1.5 mM calcium chloride with a phospholipid concentration of 27 mg/mL. Lipid enriched preparations of BLES were created through sonication at 37 °C for 2 hours. The addition of 11.6 mg or 27 mg of individual lipids (DPPC, POPC, or POPG) per mL of BLES created 30% or 50% lipid enriched versions of BLES respectively. The SP-C peptoid protein mimic (mono-SP-C) utilized in the synthetic surfactants was synthesized and purified (>97%) according to previously published protocols^24^. The lipid and peptoid compositions of the synthetic surfactant preparations are summarized in Table 1. All BLES preparations, lipid mixtures, and synthetic surfactants were used at 10 mg/mL phospholipid.

**Table 1:** Lipid and peptoid compositions for synthetic surfactants.

Surfactant Preparation	Composition of Synthetic Surfactants				
	DPPC %Phospholipids	POPC %Phospholipids	POPG %Phospholipids	Cholesterol %Phospholipids	Mono-SP-C %Weight
20% POPG	35	40	20	5	2
10% POPG	35	50	10	5	2
5% POPG	35	55	5	5	2
2.5% POPG	35	57.5	2.5	5	2
0% POPG	35	60	0	5	2
Lipids only	35	50	10	5	0

### Bacterial killing curves

Bacterial killing curves were preformed as previously reported^5^. Briefly, an overnight culture of *Pseudomonas aeruginosa* (ATCC 27853), obtained from Sigma-Aldrich (Oakville, ON, Canada), was grown in tryptic soy broth. Using measurements of optical density, 2 × 10^6^ colony forming units (CFU) of bacteria were resuspended in saline. Varying concentrations of cathelicidins 0–20 µM were then mixed with either saline (No Lipids), BLES, individual lipid components at 1–2 mg/mL phospholipid, lipid enriched BLES or a synthetic surfactant. These mixtures were then incubated with the bacteria for 3 hours at 37 °C before being serially diluted 10–10 000-fold, with 10 µl of each dilution being spot plated in triplicate on tryptic soy agar (TSA) plates. The plates were then incubated at 37 °C overnight and counted the following morning. No bacterial growth was designated as a bacterial concentration of less than 100 CFU/mL.

### Isothermal titration calorimetry (ITC)

For ITC analysis, vesicles of POPC and POPG were generated using the extrusion technique as previously reported^25^. Phospholipid content was determined as inorganic phosphate after treatment with perchloric acid by UV-VIS spectroscopy^26^. POPC and 10% POPG/90% POPC vesicles were diluted to 1.5 mg/mL, while POPG vesicles were further diluted to 0.15 mg/mL. For measurements using BLES, the stock solution was diluted to 1.5 mg/mL. Interactions between CATH-2 and large unilamellar vesicles consisting of POPC and/or POPG, or between CATH-2 and BLES were tested using ITC. All ITC experiments were performed on a Low Volume NanoITC (TA instruments - Waters LLC, New Castle, USA). In each experiment, the ITC cell chamber was filled with 190 µl of vesicles or BLES, and the syringe was filled with a 50 µl solution of 320 µM CATH-2. Titrations were incremental with 2 µl injections at 300 seconds intervals. Experiments were performed at 37 °C and data were analyzed with the Nano Analyze software (TA instruments - Waters LLC).

### Solid-State nuclear magnetic resonance (ssNMR) spectroscopy

For ssNMR, DOPG unilamellar vesicles were prepared with 5 mM HEPES pH 7.5 and 50 mM NaCl by the extrusion technique and using filters with a 0.2 µm cut off^27^. Phospholipid concentration was determined as inorganic phosphate after treatment with perchloric acid^28^. CATH-2 was added to DOPG vesicles to a final molar ratio of 1:50 CATH-2/DOPG. The interactions between CATH-2 and unilamellar vesicles of DOPG were assessed using ssNMR. Vesicles were collected after ultracentrifugation and were spun in 3.2 mm rotors. Static ^31^P ssNMR spectra were acquired at 500 MHz magnetic field (^1^H-frequency) and a sample temperature of 295 K. Heteronuclear proton decoupling did not affect the spectra and was switched off for all measurements. The resulting ^31^P powder pattern was apodised with 50 Hz exponential line-broadening and baseline corrected.

### Bacterial killing and surface tension measurements on the wet bridge transfer system

To analyze surfactant spreading and bactericidal properties, the wet bridge transfer system was set up as previously described^29^. The system consisted of a Teflon block with two 20 mm diameter wells, a delivery well, and a remote well. Each well has a depth of 1 mm and was separated by a 0.2 mm high raised Teflon bridge. A wetted piece of ashless filter paper was placed over the Teflon bridge to join the delivery and remote wells. Both wells were then filled with 1 mL of a 150 mM NaCl, 1.5 mM CaCl_2_, and 5 mM HEPES (pH 7.4) solution. To determine surfactant spreading, preparations were administered into the delivery well and surface tension was measured in the remote well using a Wilhelmy probe. FilmWare 2.51 software of the Langmuir balance was used over a period of 480-seconds after 200 µL of surfactant preparations or saline was administered to the delivery well.

For bacterial killing experiments with the lipid enriched versions of BLES, the overnight culture of *P. aeruginosa* was serially diluted to 2 × 10^5^ CFU and seeded to the remote well. Then 200 µL of cathelicidins (0–100 µM) were administered to the surface of the delivery well with saline, BLES or 30% lipid enriched BLES preparations. For bacterial killing experiments with the synthetic surfactant preparations, the wet bridge transfer system was modified through the addition of a lower sucrose layer. Both wells were filled with 800 µL of a 10% sucrose solution (150 mM NaCl, 1.5 mM CaCl_2_, 5 mM HEPES). Then 100 µL of the same solution without sucrose was added to make a thin upper layer on top of the sucrose layer. For these experiments the overnight culture of *P. aeruginosa* was serially diluted to 2 × 10^6^ CFU of bacteria and seeded to the remote well above the sucrose layer. For treatments, 100 µL of either saline, BLES or synthetic surfactants with or without CATH-2 (100 µM) were administered to the surface of the delivery well. Eight minutes after administration the wetted piece of filter paper was removed, and all fluid was collected from both dishes to be incubated at 37 °C for 3 hours. Then 50 µL of each sample from the remote well was diluted with 50 µL of saline in a polypropylene coated 96-well plate and subsequently diluted 10–10,000-fold. CFU/mL was determined by spot plating 10 µL of each dilution in triplicate on TSA plates. These plates were incubated overnight at 37 °C and colonies were counted to a detection limit of 100 CFU/mL. Samples from the delivery dish were also spot plated on TSA plates and incubated overnight at 37 °C to ensure no bacterial transfer.

### Statistical analysis

All data points shown represent the average of at least three independent repetitions. Statistical significance was determined by two-way analysis of variance and one-way analysis of variance (ANOVA) followed by a Tukey-Kramer post hoc test to determine differences among experimental groups. Results are presented as mean ± the standard deviation and were considered statistically significant with a P-value of less than 0.05.

## Results

### Bacterial killing curves

**Figure 1:**
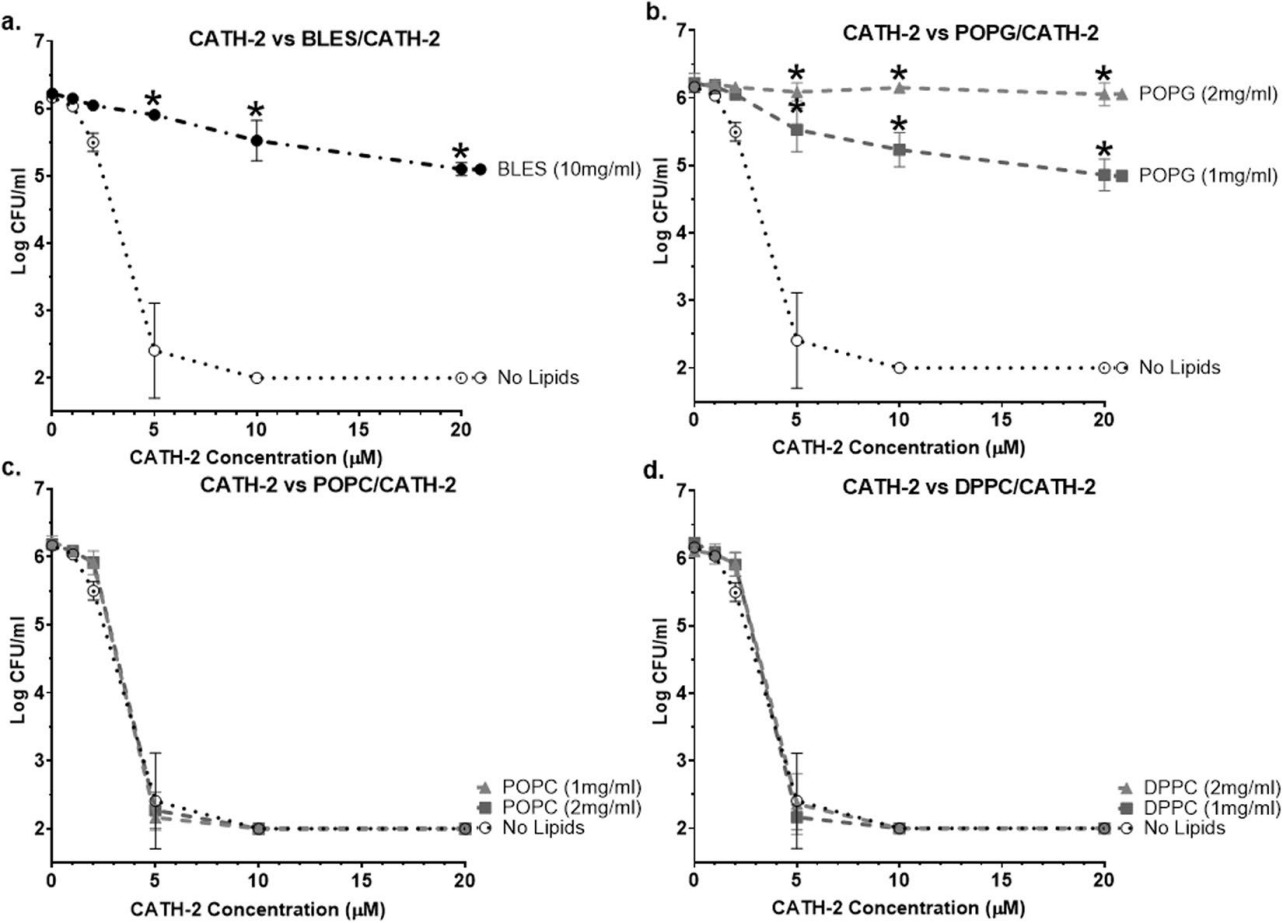
Killing curves for CATH-2 combined with BLES or individual surfactant lipids. Shown are the bacterial killing curves for CATH-2 suspended in either A) BLES, B) POPG, C) POPC, D) DPPC or No Lipids (saline). Colonies were counted to a detection limit of 100 CFU/mL. *p < 0.05 vs No Lipids. Error bars = SD; n = 3.

To investigate how exogenous surfactant and its lipid components affect antimicrobial peptide function, bacterial killing curves were performed with CATH-2 combined with 10 mg/mL BLES or 1–2 mg/mL of individual surfactant lipids (Fig. 1). Shown on each of the panels (Fig. 1A–D) for comparison purposes, CATH-2 combined with saline (No Lipids) exhibited potent bactericidal activity against *P. aeruginosa*, reducing bacterial growth below detectable limits at 5 to 10 μM. In the presence of 10 mg/mL BLES, the antimicrobial properties of CATH-2 were significantly reduced at concentrations of 5 μM or greater (Fig. 1A). Since BLES contains approximately 10% PG, CATH-2 mediated killing was tested in the presence of 1 mg/mL POPG. Similar to BLES, the presence of POPG at 1 mg/mL phospholipid significantly reduced CATH-2 killing at concentrations of 5 μM or greater compared to the peptide combined with no lipids (Fig. 1B). Additionally, mixing CATH-2 with 2 mg/mL POPG resulted in a complete loss of its bactericidal properties. In contrast to the effect of POPG, there was no significant difference in the bacterial killing of CATH-2 combined with DPPC or POPC at 1–2 mg/mL phospholipid, compared to the peptide with no lipids (Fig. 1C,D). To explore if the PG effect was specific to CATH-2, killing curves for CRAMP, PMAP-23 and LL-37 were also performed in the presence of BLES or individual surfactant lipids. All three peptides showed complete inhibition of their antimicrobial function when combined with 10 mg/mL BLES or 1–2 mg/mL POPG (see Supplemental Fig. S1).

**Figure 2:**
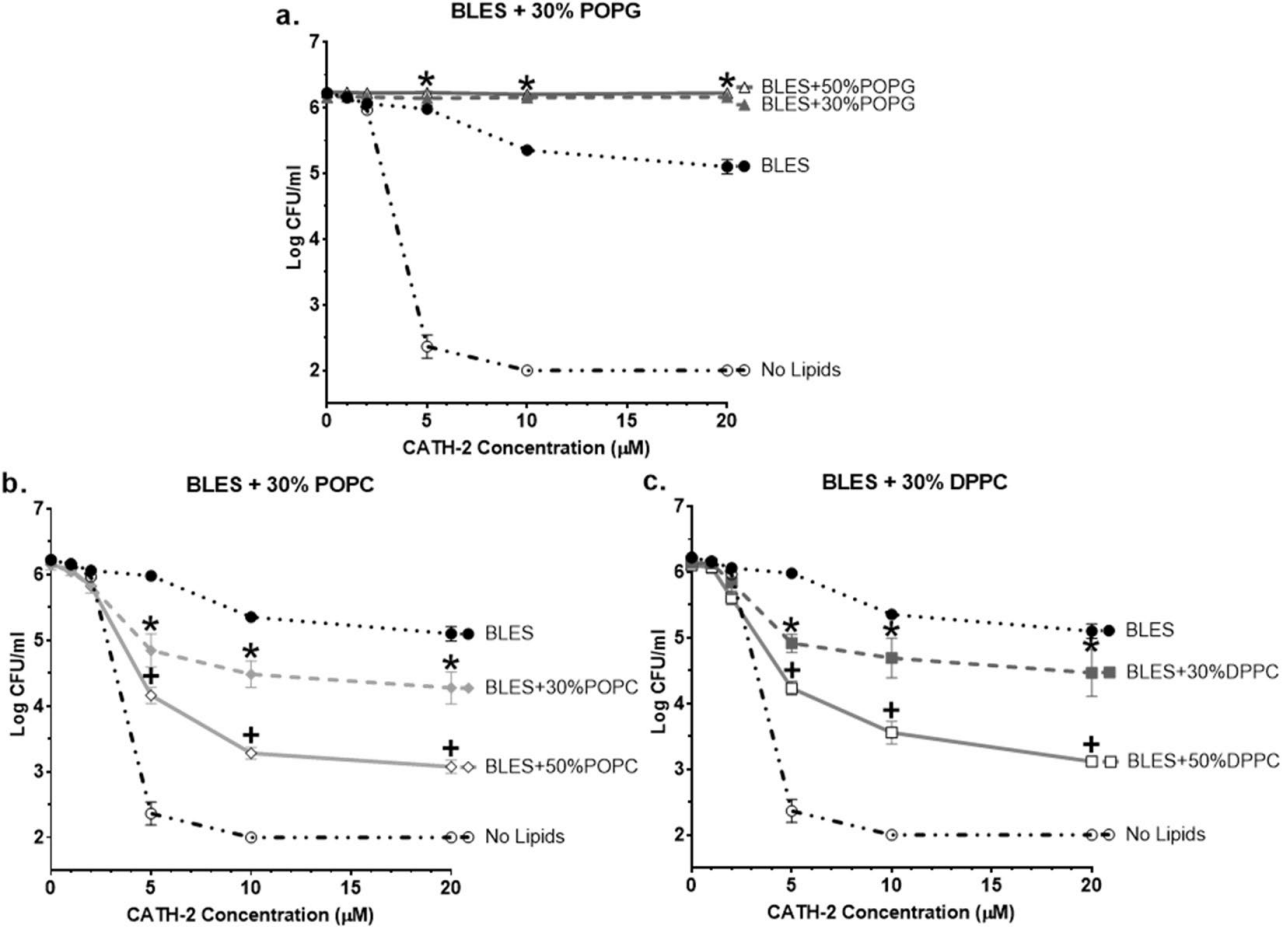
Antimicrobial activity of CATH-2 in lipid enriched preparations of BLES. Displayed are the bacterial killing curves for CATH-2 suspended in 10 mg/mL of 30–50% A) POPG, B) POPC, or C) DPPC enriched versions of BLES. Colonies were counted to a detection limit of 100 CFU/mL. *p < 0.05 for BLES + 30% lipid vs BLES, ^+^p < 0.05 for BLES + 30% lipid vs BLES + 50% lipid. Error bars = SD; n = 3.

To determine the effects of modifying an existing surfactant’s lipid composition on cathelicidin function, bacterial killing curves were performed for CATH-2 mixed with 30–50% lipid enriched preparations of BLES (10 mg/mL). Suspension of CATH-2 in 30% or 50% POPG enriched BLES resulted in a complete loss of the peptide’s antimicrobial properties (Fig. 2A). However, at concentrations of CATH-2 of 5 μM or greater, the 30% DPPC and POPC enriched BLES preparations were found to kill significantly more bacteria than BLES/CATH-2, resulting in a 2–log reduction in bacterial viability at 20 μM (Fig. 2B,C). At those concentrations of CATH-2, 50% DPPC or POPC diluted BLES also showed significantly more bacterial killing compared to their 30% equivalents.

### ITC and ssNMR of CATH-2 mixed with individual surfactant lipids

**Figure 3:**
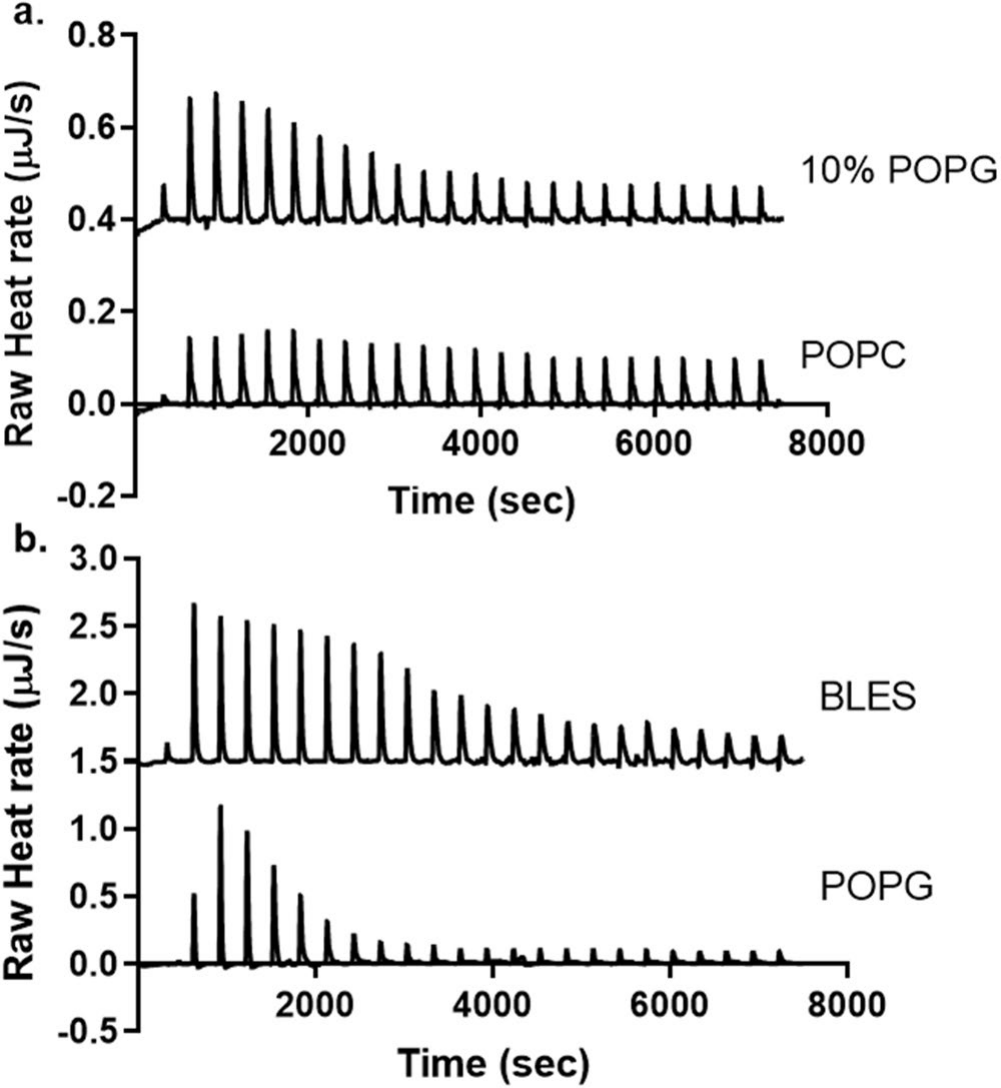
ITC binding of CATH-2 to vesicles and BLES. Shown are representative thermograms of ITC experiments with titration of 320 μM CATH-2, into 1.5 mg/mL BLES, POPC and 10% POPG vesicles, or 0.15 mg/mL POPG vesicles.

To further examine the interactions between cathelicidins and exogenous surfactant, CATH-2 and large unilamellar vesicles of individual lipids or BLES were tested using ITC. As displayed in Fig. 3A (bottom panel), there was no, or very little heat production observed when CATH-2 was injected into the POPC sample, with an enthalpy of -3.0 kJ/mol. However, when 10% POPG was added to the vesicles, a large increase in heat production was observed following CATH-2 injection, indicative of exothermic binding (Fig. 3A; top panel; ΔH = -13.2 kJ/mol). For both BLES and 100% POPG vesicles, the binding of CATH-2 resulted in a 3–fold higher release of heat (Fig. 3B), with enthalpies of -34.6 and -34.2 kJ/mol respectively.

**Figure 4:**
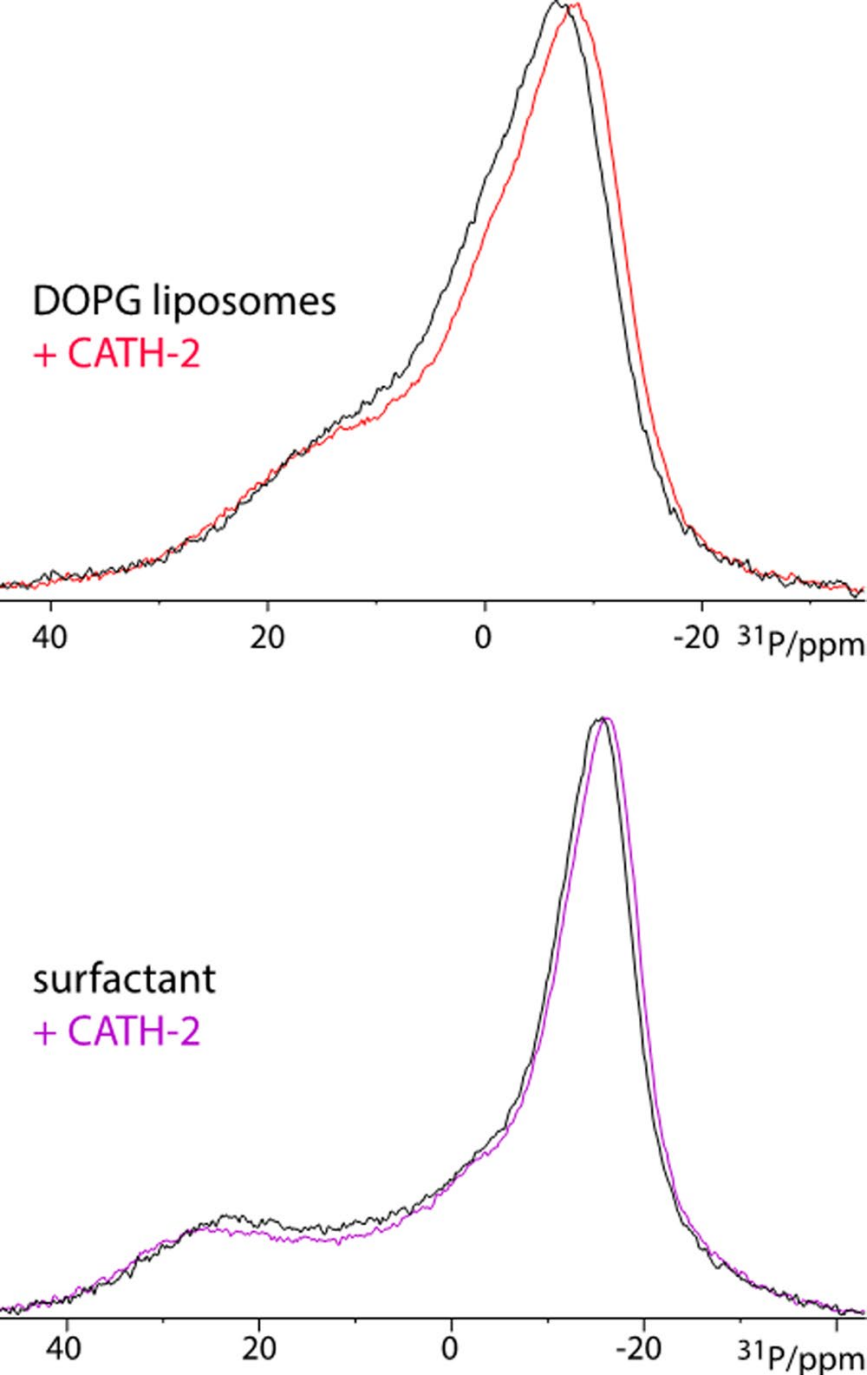
ssNMR of CATH-2 mixed with BLES or liposomes. Static ^31^P solid-state NMR spectra acquired at 500 MHz magnetic field. *(upper panel)* Spectra acquired with unilamellar DOPG vesicles in the absence (black lines) or presence of CATH-2 (red lines). The span of the powder pattern at approximately 10% signal height is 42.6 ppm (8610 Hz) and 41 ppm (8300 Hz) in the presence and in the absence of CATH-2, respectively. *(lower panel)* Spectra were acquired with BLES surfactant in the absence (black lines) and the presence of CATH-2 (magenta lines). The span of the powder pattern at approximately 10% signal height is 59.7 ppm (12090 Hz) and 57.1 ppm (11560 Hz) in the presence and in the absence of CATH-2, respectively. All spectra are normalised.

With ssNMR spectroscopy, CATH-2 combined with unilamellar vesicles of DOPC and DOPG were assessed by acquiring ^31^P chemical shift anisotropy powder pattern spectra under static conditions. These patterns result solely from the ^31^P chemical shift anisotropy and are sensitive to both the lipid headgroup mobility and orientation^30–32^. The addition of CATH-2 to the DOPG vesicles resulted in a modest but clear broadening of the ^31^P powder pattern by 1.6 ppm (310 Hz) (Fig. 4). For BLES, the addition of CATH-2 caused a broadening of the power pattern by 2.6 ppm (470 Hz), similar as in DOPG, demonstrating peptide-surfactant binding and modulation of the surfactant headgroups. Lastly, isotropic signals were not observed for any of the measurements, indicating that CATH-2 does not cause very strong curvature to the membrane or the formation of spherical micelle-like structures (while curvature effects could still modulate the ^31^P powder spectrum)^32,33^.

### Bacterial killing and spreading over the wet bridge for lipid enriched BLES

**Figure 5:**
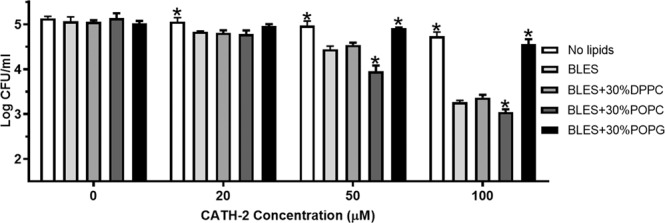
Bacterial killing for CATH-2 over the wet bridge transfer system. Presented are the bacterial counts in the remote dish (2 × 10^5^ CFU/mL *P. aeruginosa* seeded) three hours following the administration of CATH-2, suspended in saline (No lipids), BLES, or 10 mg/mL 30% lipid enriched versions of BLES, to the delivery dish. *p < 0.05 vs BLES. Error bars = SD, n = 3.

To further examine the effect of PG on CATH-2 responses, BLES preparations were enriched with individual lipids to change the relative percentage of PG within the preparation. POPC and DPPC at 30% and 50% were utilized to decrease the relative PG content, whereas POPG was used to increase the PG content within the BLES preparation. Subsequently, the wet bridge transfer system was utilized to investigate if CATH-2, suspended in lipid enriched preparations of BLES, could affect bacterial killing, at a distal site. Suspension in BLES resulted in significantly more bacterial killing by CATH-2 (20–100 µM) in the remote well, compared to the peptide alone (Fig. 5). CATH-2 (50–100 µM) suspended in 30% POPC enriched BLES resulted in significantly more bacterial killing compared to BLES/CATH-2. When mixed with 30% DPPC enriched BLES, CATH-2 (0–100 µM) displayed no change in bacterial killing, compared to BLES/CATH-2. Additionally, the suspension of CATH-2 (50–100 µM) in 30% POPG enriched BLES resulted in significantly less bacterial killing at the remote dish compared to BLES/CATH-2. Suspending the other AMPs, CRAMP, PMAP-23, or LL-37 at 100 µM with 30% POPC enriched BLES were also shown to significantly improve bacterial killing in the remote well compared to BLES/peptide or the peptide with no lipids (see Supplemental Fig. S2).

**Figure 6:**
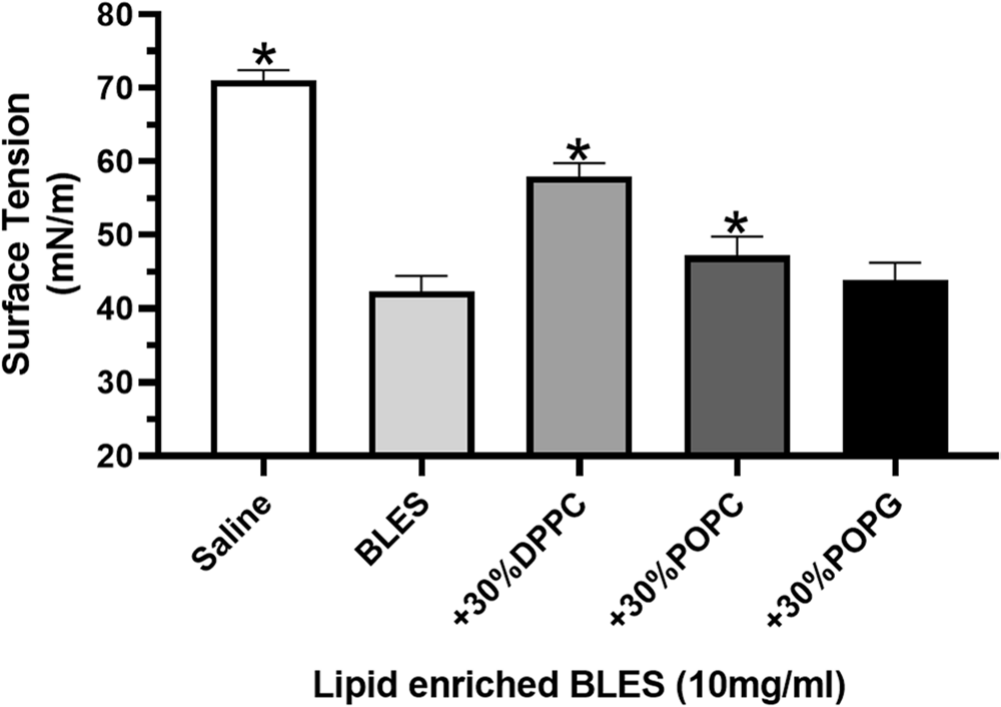
Surfactant spreading over the wet bridge transfer system for lipid enriched BLES. Shown are the surface tensions achieved in the remote dish after the 480-second period following administration of saline, BLES (10 mg/mL) or 30% lipid enriched preparations of BLES to the delivery dish. *p < 0.05 vs BLES. Error bars = SD; n = 4.

To examine the spreading characteristics of the 30% lipid enriched preparations of BLES, the surface tension in the remote well of the wet bridge was measured over a 480-second period after instillation into the delivery well. All 30% lipid modified mixtures of BLES achieved surface tensions significantly lower than saline (Fig. 6). However, the surface tension achieved by 30% DPPC or POPC diluted BLES were significantly higher than BLES alone.

### Bacterial killing curve for synthetic surfactants

**Figure 7:**
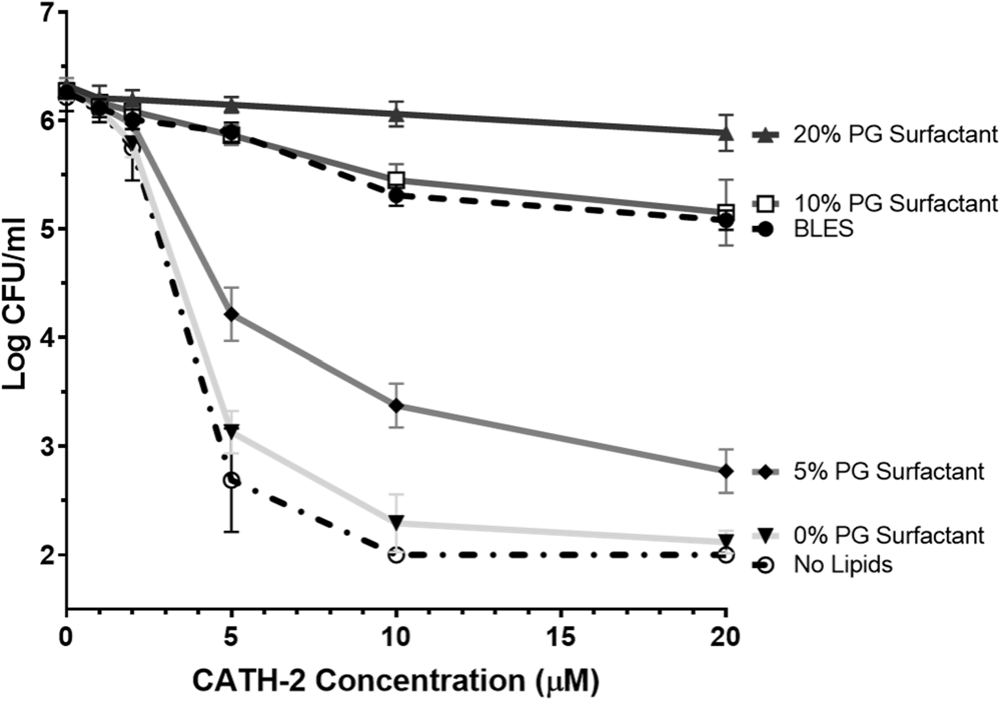
Bacterial killing curves for CATH-2 combined with synthetic surfactants. Shown are the bacterial killing curves for CATH-2 suspended in saline (No Lipids), 10 mg/mL of BLES or synthetic surfactants with varying PG content. Colonies were counted to a detection limit of 100 CFU/mL. The composition of all synthetic surfactants is displayed in Table 1. Error bars = SD; n = 3.

To further explore the inhibitory effects of PG and the potential of a PG-free surfactant, bacterial killing curves were performed for CATH-2 combined with synthetic surfactants of varying PG content. At CATH-2 concentrations of 5 μM and above, bacterial killing was found to be significantly greater for synthetic surfactants with 5% or less POPG and significantly lower for preparations with 20% POPG compared to BLES (Fig. 7). The synthetic surfactant with a POPG composition of 10% displayed similar bactericidal properties to BLES when combined with CATH-2 at 0–20 μM. Lastly, combining CATH-2 with a PG-free synthetic surfactant (0% POPG) resulted in antimicrobial properties no different than the peptide alone.

### Bacterial killing and spreading over the wet bridge for synthetic surfactants

**Figure 8:**
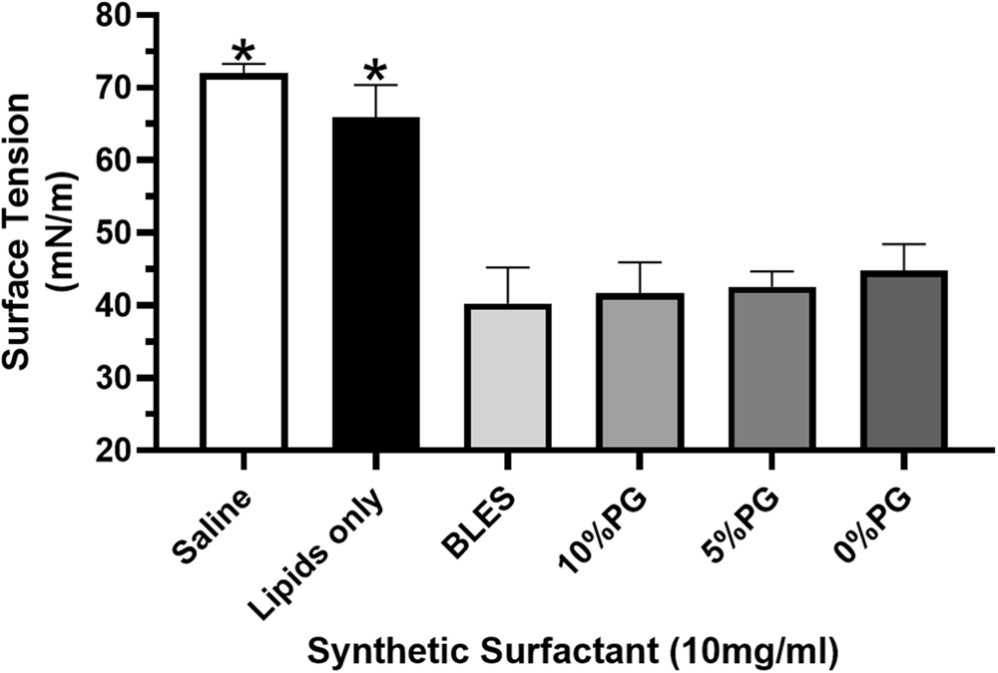
Spreading over the wet bridge transfer system for synthetic surfactants. Shown are the surface tensions achieved in the remote dish after the 480-second period following administration of saline, lipids only, BLES (10 mg/mL) or a synthetic surfactant preparation to the delivery dish. *p < 0.05 vs BLES. Error bars = SD; n = 4.

To examine the spreading characteristics of the synthetic surfactants, the surface tension in the remote well of the wet bridge was measured over a 480-second period after instillation into the delivery well. All synthetic surfactants tested achieved surface tensions similar to BLES and significantly lower than saline or the lipids alone (Fig. 8).

**Figure 9:**
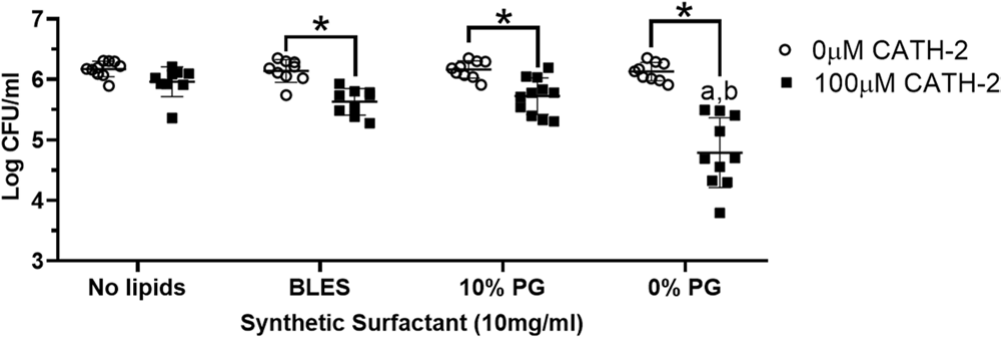
Bacterial killing over the wet bridge for synthetic surfactants/CATH-2. Presented are the bacterial counts in the remote dish (2 × 10^6^ CFU/mL P. aeruginosa seeded) three hours following administration to the delivery dish of either saline (No lipids), BLES, or synthetic surfactants with or without CATH-2. ^a^p < 0.05 vs BLES, ^b^p < 0.05 vs 10% PG synthetic surfactant, *p < 0.05. Error bars = SD; n = 9–11.

To further evaluate the efficacy of a PG-free synthetic surfactant for delivering AMPs to a distal site, CATH-2 (100 µM) was administered to the deliver dish of the wet bridge transfer system with no lipid, BLES or in combination with a synthetic surfactant. Administering CATH-2 with BLES or the 10% PG synthetic surfactant showed similar bacterial killing at the remote well (Fig. 9). CATH-2 suspended in a PG-free synthetic surfactant resulted in significantly more bacterial killing in the remote well than all other preparations mixed with the peptide. This data implies that the synthetic surfactant without PG was capable of spreading over the wet bridge and acting as a carrier for CATH-2 to induce killing at a remote location.

## Discussion

The current study tested the hypothesis that the PG component of surfactant inhibits CATH-2 function and that an exogenous surfactant with a reduced PG composition would increase peptide mediated killing at distal sites. Overall, our results supported this hypothesis. Specifically, measurements of bacterial killing as well as both assessments of binding indicated that PG interacts with CATH-2, inhibiting its function. Furthermore, reducing or eliminating the PG content in an exogenous surfactant improved the ability of CATH-2 to kill bacteria. It was also shown that a PG-free synthetic surfactant was capable of carrying CATH-2 to distal sites to kill bacteria. Based on these observations it is concluded that synthetic PG-free surfactants will enhance the pulmonary delivery of CATH-2 without inhibiting its antimicrobial function.

Clinically, the relevance of this study is associated with the potential positive benefits of treating bacterial lung infections with cathelicidins delivered by a surfactant vehicle to reach the deeper areas of the lung. For many pneumonia patients, the acquisition of antimicrobial-resistant bacteria represents a decisive stage in disease progression, towards poor outcomes^34–38^. The ability of CATH-2 and other AMPs to target antibiotic-resistant bacteria make these molecules interesting for the development of novel therapeutics^11,39,40^. The ability of exogenous surfactants, to re-open collapsed airways and allow antimicrobials access to regions blocked off during the infection will also be essential for combating resistance^41–43^. The direct delivery of antimicrobials will elevate local therapeutic concentrations at the pulmonary sites of infection, improving bacterial clearance and limiting the development of resistance^15,44^. Together, CATH-2 and exogenous surfactant represent a desperately needed treatment strategy to address the growing threat of multidrug-resistant lung infections.

An important aspect of the current paper was the utilization of a synthetic PG-free surfactant, customized for its ability to maintain CATH-2 activity. Several molecular dynamic simulations have suggested that cathelicidins would electrostatically bind the anionic lipids in bacterial membranes^45–48^. Similarly, NMR studies have also demonstrated that AMPs have a strong tendency to form helical structures that bind negatively charged molecules, such as the lipopolysaccharides of Gram negative bacteria^49,50^. Altering such electrostatic interactions, through additional anionic lipids, lipid lysinylation, or changes to the cationic nature of the peptide, have all been shown to alter bacterial killing^51–54^. In support of these observations, our ssNMR, ITC and bacterial killing data provides strong evidence that PG was the inhibitory component of the exogenous surfactant, likely by restricting CATH-2 from directly or indirectly binding negatively charged lipids, DNA or other bacterial components^11,39,55^. Moreover, the enhancement in CATH-2 function following the addition of POPC or DPPC to BLES, illustrates the potential benefit for lowering PG content in surfactant to reduce electrostatic interactions with these peptides. As such, we generated a PG-free surfactant using a surfactant protein mimic of SP-C and demonstrated that such a surfactant, mixed with CATH-2, was significantly better at killing bacteria at a remote site compared to PG-containing surfactants. Since AMPs have been shown to form weak hydrophobic interactions with neutral lipid membranes^56–58^ it is likely that the PG-free preparation transported CATH-2 via these weaker hydrophobic interactions. Importantly, this weaker binding likely allowed CATH-2 to still interact with the bacteria at the remote site. Thus, we conclude that synthetic PG-free surfactants are optimal delivery vehicles for CATH-2 and that it is worthwhile investigating such therapeutics in future *in vivo* studies.

The generation of a PG-free surfactant for the delivery of CATH-2 also provided proof of a more general concept, that synthetic surfactants can be customized for drug delivery. The majority of studies exploring surfactant as a carrier of pulmonary antibiotics and other pulmonary therapeutics simply mix the drug with a commercial exogenous surfactant preparation developed for the treatment of surfactant deficient premature infants rather than as a delivery vehicle^3,16,29,41,59^. Although some success has been obtained with these approaches^3^, many of the surfactants utilized were animal derived preparations in which the composition is established by its endogenous source. The advantage of a synthetic surfactant is that the specific composition can be optimized for the delivery of drugs. We utilized an approach of using surfactant protein mimics, or peptoids, since a recent study demonstrated that these peptoid-based surfactant were not only active *in vivo*, but equivalent to animal-derived surfactants for improving oxygenation and other physiological outcomes in a model of acute lung injury^60^. Peptoids are structurally based on a polypeptide backbone but with side-chains appended to nitrogen-backbone, they are highly stable to proteolysis and can be made in high yields^24,61,62^. Once synthesized, the peptoids can also be easily mixed with a specific lipid mixture optimized to spread throughout the lung and for drug delivery, as illustrated *in vitro* with our PG-free surfactant. Overall, we propose that the development of peptoids and other synthetic analogs of the hydrophobic surfactant proteins will allow for a more mechanistic approach to developing surfactant-based drug delivery approaches.

Having established the effect of exogenous surfactant on CATH-2, there is also therapeutic value in exploring if similar approaches can be utilized for other cathelicidins, thereby increasing the clinical arsenal of antimicrobial agents. Unfortunately, the presence of exogenous surfactant completely abolished the antimicrobial function of many cathelicidins, including human LL-37, mouse CRAMP and pig PMAP-23^8^. However, the current study combined each of these peptides with a lipid enriched preparation of surfactant and demonstrated that they could all benefit from delivery by a surfactant with a reduced PG content. This ability to create functional exogenous surfactants, that minimally interact with a variety of AMPs, will substantially increase the treatment options for lung infections. For these reasons, developing exogenous surfactant as a delivery vehicle for multiple AMPs, each with their own unique antibacterial pathway, would create a promising new pipeline of anti-infective therapeutics.

It should also be noted that there are several limitations to our study. First, the bacterial killing was limited to one strain of bacteria. However, it is important to note that our previous study showed a similar pattern of inhibition for CATH-2, by surfactant against several Cystic Fibrosis derived bacterial strains. As such, we anticipate that PG-free surfactant with CATH-2 will likely provide improved killing activity against other strains as well^5^. Secondly, the PG-free synthetic surfactant was only tested in combination with one cathelicidin, CATH-2. However, we did demonstrate that several other cathelicidins were also inhibited by PG and benefited from a lipid enriched surfactant with a reduced PG composition. From these findings, we predict that LL-37, CRAMP, and PMAP-23 would all benefit from delivery by a PG-free synthetic surfactant. It should also be noted that high concentrations of AMPs have been associated some cytotoxic effects towards mammalian cells, potentially limiting their therapeutic potential^8,63–65^. However, a recent *in vivo* study showed that co-instillation of CATH-2 with an exogenous surfactant was well tolerated, with no deleterious effects up to 100 µM^8^. Lastly, our synthetic PG-free surfactant was only designed based on minimizing PG content. The preparation contains POPC, DPPC and cholesterol as well as an SP-C peptoid, however, this composition could be further optimized with respect to these remaining components as well as other surfactant components.

In conclusion, this paper has demonstrated how exogenous surfactant can be designed to be a more effective delivery system for CATH-2. Further, we propose that this concept could be applied to other intrapulmonary therapeutics. Direct drug delivery is a major hurdle for many pulmonary conditions and designing exogenous surfactants with specific drug-delivery properties offers an intriguing method to overcome that obstacle.

## Supplementary information

Supplementary information.
