# Supplementary material for: Optimizing Exogenous Surfactant as a Pulmonary Delivery Vehicle for Chicken Cathelicidin-2
Source: Sci Rep. 2020 Jun 10;10:9392. doi: 10.1038/s41598-020-66448-1 (PMC7287084; doi:10.1038/s41598-020-66448-1)
Supplement: Supplementary file 1 — Supplementary information. [file 41598_2020_66448_MOESM1_ESM.docx]

**Optimizing Exogenous Surfactant as a Pulmonary Delivery Vehicle for Chicken Cathelicidin-2**

Brandon Baer,^a^ Edwin J. A. Veldhuizen,^b^ Natalia Molchanova,^c,d^ Shehrazade Jekhmane,^e^ Markus Weingarth,^e^ Håvard Jenssen,^c^ Jennifer S. Lin,^f^ Annelise E. Barron,^f^ Cory Yamashita,^a,g^ Ruud Veldhuizen^a,g^

^a^Department of Physiology and Pharmacology, Western University, London, Ontario, Canada

^b^Department of Infectious Diseases and Immunology, Faculty of Veterinary Medicine, Utrecht University, Utrecht, The Netherlands

^c^Department of Science and Environment, Roskilde University, Roskilde, Denmark

^d^Division of Biological Nanostructures, The molecular Foundry, Lawrence Berkeley National Laboratory, Berkeley, California, USA

^e^Bijvoet Center for Biomolecular Research, Department of Chemistry, Utrecht University, Utrecht, The Netherlands

^f^Department of Bioengineering, School of Medicine & School of Engineering, Stanford University, Stanford, California, USA

^g^Department of Medicine, Western University, London, Ontario, Canada

**Fig. S1. Bacterial killing for AMPs combined with individual surfactant lipids.**

Shown are the bacterial killing curves for A) PMAP-23, B) LL-37, or C) CRAMP suspended in saline (No Lipids), BLES, POPG, POPC, or DPPC against 2x10^6^ CFU/mL *P. aeruginosa*. Error bars = SD; n=3. Porcine PMAP-23, human LL-37 and mouse CRAMP were all synthesized and purified as described previously (1). Bacterial colonies were counted to a detection limit of 100 CFU/mL. When combined with no lipids each of the AMPs exhibited potent bactericidal activity. However, in the presence of BLES or 1–2 mg/mL POPG the antimicrobial properties of all three cathelicidins were completely abolished. There was no difference in the bacterial killing of any of the AMPs in the presence of DPPC or POPC at 1–2 mg/mL phospholipid compared to the peptide with no lipids.

**Fig. S2. Bacterial killing for cathelicidins over the wet bridge transfer system.**

Presented are the bacterial counts in the remote well (2x10^5^ CFU/mL *P. aeruginosa* seeded) three hours following administration to the delivery well of either saline (No Lipids), BLES (10 mg/mL) or 30% lipid enriched versions of BLES (10 mg/mL) with or without CRAMP, PMAP-23, or LL-37 at 100µM. *p<0.05 vs BLES. Error bars = SD, n=3. When combined with 30% POPC enriched BLES all three peptides killed significantly more bacteria in the remote well compared to BLES or no lipids.

**Supplementary Figure S1**

**Supplementary Figure S2**

References

1. Bikker FJ, Kaman-van Zanten WE, de Vries-van de Ruit A-MBC, Voskamp-Visser I, van Hooft P a V, Mars-Groenendijk RH, de Visser PC, Noort D. 2006. Evaluation of the antibacterial spectrum of drosocin analogues. Chem Biol Drug Des 68:148–153.
